# Acute Myocarditis Following Zoledronic Acid Infusion: Cardiac MRI Diagnosis of a Rare Cardiotoxic Event with Contextual Literature Review

**DOI:** 10.3390/diagnostics15162064

**Published:** 2025-08-18

**Authors:** Ismaell Massalha, Reem Zabit, Aryeh Shalev, Gal Ben-Arie

**Affiliations:** 1 The Legacy Heritage Center & Dr. Larry Norton Institute, Soroka Medical Center, Ben Gurion University, Beer Sheva 84105, Israel; 2 Department of Oncology, Ziv Medical Center, Safed 13100, Israel; 3 Department of Pediatrics, Soroka Medical Center, Ben Gurion University, Beer Sheva 84105, Israel; zabit@post.bgu.ac.il; 4 Department of Radiology, Soroka Medical Center, Ben Gurion University, Beer Sheva 84105, Israel; aryehsh@clalit.org.il (A.S.); galben@bgu.ac.il (G.B.-A.)

**Keywords:** zoledronic acid, myocarditis, cardiac MRI, drug-induced cardiotoxicity, bisphosphonates

## Abstract

Background and Clinical Significance: Zoledronic acid (ZA) is a widely used bisphosphonate for the prevention of skeletal-related events in patients with metastatic bone disease. While it is generally well tolerated, rare immune-mediated complications may be underrecognized. To date, myocarditis has not been reported in association with ZA. Case Presentation: A 35-year-old woman with metastatic pheochromocytoma developed acute, non-exertional chest pain approximately 36 h after receiving her first intravenous ZA infusion. Laboratory testing revealed elevated high-sensitivity troponin T, peaking at 1182 ng/L. Cardiac magnetic resonance imaging (CMR) demonstrated myocardial edema and subepicardial late gadolinium enhancement, consistent with acute myocarditis per the 2018 revised Lake Louise criteria. An extensive diagnostic workup excluded infectious, autoimmune, and ischemic causes. Symptoms and troponin levels improved following ZA discontinuation and supportive care. In the absence of alternative etiologies, and given the close temporal association with ZA administration, the diagnosis of presumed ZA-associated myocarditis was supported by clinical presentation, biochemical markers, and CMR findings, recognizing that histopathological confirmation is rarely pursued in clinically stable patients. Conclusions: To our knowledge, this is the first reported case of presumed zoledronic acid–associated myocarditis confirmed by cardiac MRI. This report highlights the diagnostic utility of CMR in suspected drug-related cardiac inflammation and the importance of considering myocarditis in patients presenting with unexplained chest pain following ZA infusion, particularly when other causes have been excluded.

## 1. Introduction

Zoledronic acid (ZA) is a widely utilized nitrogen-containing bisphosphonate primarily employed to reduce skeletal-related events in patients with metastatic bone disease, osteoporosis, and malignancy-associated hypercalcemia [1,2]. By inhibiting osteoclast-mediated bone resorption, ZA significantly reduces the risk of pathological fractures and other skeletal complications, particularly in patients with advanced malignancies. Due to its efficacy and relatively favorable safety profile, ZA remains a cornerstone in the management of bone metastases and related complications [3,4,5,6].

Despite its therapeutic benefits, ZA is not devoid of adverse effects. Commonly reported complications include acute-phase reactions, nephrotoxicity, atrial fibrillation, and hypocalcemia [7]. Additionally, emerging data indicate that ZA may provoke immune-mediated responses, leading to systemic inflammatory syndromes, sepsis-like presentations, and metabolic myopathies [8]. Although these reactions are uncommon, their occurrence remains clinically significant, especially given ZA’s extensive use in oncology and endocrinology.

One area of particular interest, yet insufficiently explored, is the potential link between ZA and myocarditis, an inflammatory cardiac condition characterized by myocardial infiltration by immune cells, often resulting in myocardial edema, necrosis, and fibrosis. Myocarditis can have various etiologies, including viral infections, autoimmune responses, and drug reactions. Drug-induced myocarditis presents a diagnostic challenge due to its nonspecific symptoms and absence of definitive biomarkers. As histopathological confirmation is rarely feasible in stable patients, the diagnosis of myocarditis typically relies on a combination of clinical presentation, laboratory findings, and advanced imaging modalities—particularly cardiac MRI (CMR). Utilizing the Lake Louise criteria, CMR enables the non-invasive detection of myocardial inflammation through indicators such as increased myocardial T2 signal intensity (reflecting edema) and late gadolinium enhancement (LGE), which is suggestive of myocardial edema, necrosis, and fibrosis [9].

While myocarditis has been extensively documented with immune checkpoint inhibitors (ICIs) and certain chemotherapeutic agents [10,11], where immune activation triggers myocardial inflammation, reports linking bisphosphonates, particularly ZA, to myocarditis remain sparse. Nonetheless, mechanistic studies and case reports have suggested that ZA may modulate inflammatory cytokines, particularly TNF-α, IL-1β, and IL-6, through activation of the NLRP3 inflammasome and immune pathways in a manner conducive to myocardial inflammation [12], potentially via effects on electrolyte balance and pro-inflammatory signaling [7,13]. Such mechanistic insights, while compelling, require cautious interpretation given the lack of direct clinical evidence linking ZA to myocarditis.

Despite its theoretical plausibility, the clinical manifestation of ZA-induced myocarditis remains exceedingly rare. Documented cases are few, with one report describing acute myopericarditis following intravenous ZA in a patient with Paget’s disease, resolving upon discontinuation [9]. Additional reports have documented sepsis-like syndromes and myocardial dysfunction shortly after ZA administration [8], underscoring the immunologic underpinnings of these rare complications.

In this context, we present a unique case of presumed acute myocarditis temporally associated with the first ZA infusion in a patient with metastatic pheochromocytoma. The diagnosis was supported by clinical findings, laboratory evidence of myocardial injury, and confirmation on CMR. To our knowledge, this represents the first instance of CMR-confirmed myocarditis temporally associated with ZA administration. While causality cannot be definitively established without histopathological confirmation, the temporal proximity and imaging findings warrant clinical attention.

Given the widespread use of ZA, awareness of its potential, albeit rare, cardiac complications is critical. Clinicians should maintain a high index of suspicion for myocarditis in patients presenting with acute chest pain following ZA therapy, especially when initial evaluations are inconclusive. Early recognition and discontinuation may mitigate adverse outcomes. This case further emphasizes the value of CMR in diagnosing atypical cardiac presentations and calls for continued pharmacovigilance and research to clarify the incidence, pathophysiology, and risk factors associated with ZA-induced myocarditis.

## 2. Case Presentation

A 35-year-old woman presented to the emergency department with intermittent, non-exertional chest discomfort approximately 36 h after receiving her first intravenous infusion of ZA for metastatic bone disease. The pain was described as a dull ache localized to the mid-anterior chest, without positional or pleuritic characteristics. The patient denied associated symptoms such as dyspnea, palpitations, or syncope. Upon admission, she was afebrile, with stable vital signs, and her physical examination was unremarkable.

### 2.1. Medical History

**Figure 1:**
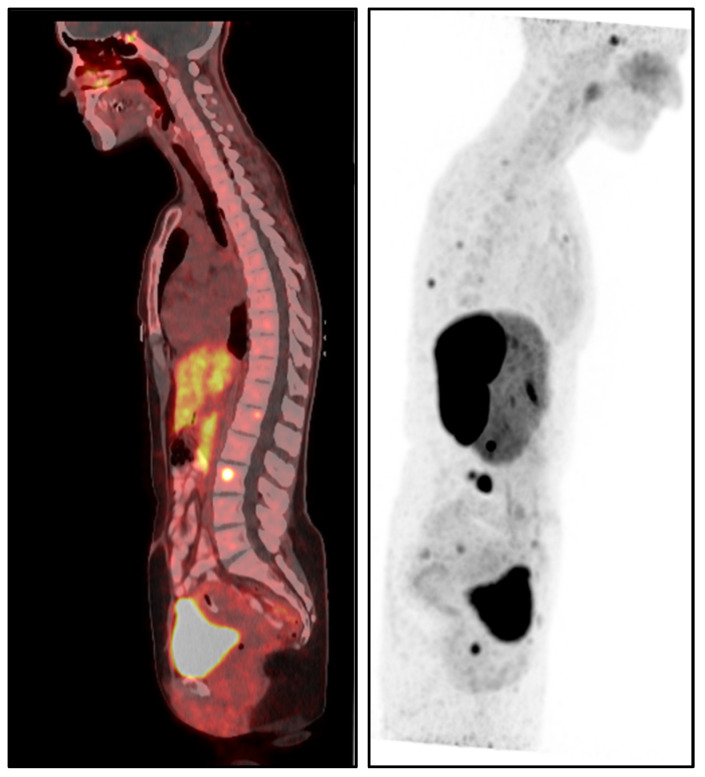
DOPA PET/CT sagittal images showing increased radiotracer uptake in the skeletal system, consistent with skeletal metastases from metastatic pheochromocytoma. The images demonstrate significant involvement in the spine and pelvis.

The patient’s medical history was notable for metastatic pheochromocytoma, initially managed with a left adrenalectomy in 2017. After surgery, she remained under routine surveillance until August 2022, when she was diagnosed with skeletal metastasis confirmed by dihydroxyphenylalanine positron emission tomography/computed tomography (DOPA PET/CT) (Figure 1). Since the diagnosis, her disease has been managed with monthly somatostatin analog therapy (Lanreotide 120 mg), and she has remained in stable condition. Additionally, she had a history of papillary thyroid carcinoma (stage I, T1aN0M0), treated with right partial thyroidectomy in 2021, and was maintained on levothyroxine therapy. The patient had no history of smoking, cardiovascular disease, or autoimmune conditions.

### 2.2. Clinical Timeline and Diagnostics

Following ZA infusion, the patient reported the onset of chest pain, initially described as abdominal discomfort that progressively radiated to the chest and scapular region. Approximately 36 h after symptom onset, she developed palpitations, weakness, and a choking sensation, prompting presentation to the emergency department.

On arrival, the patient was hemodynamically stable, afebrile, and in no apparent distress. Electrocardiography (ECG) demonstrated sinus rhythm with mild ST segment depressions in the chest leads, without significant arrhythmias or conduction abnormalities. Transthoracic echocardiography (TTE) revealed the following:

- Mild left ventricular systolic dysfunction (ejection fraction 45%).
- Mild-to-moderate mitral regurgitation.
- Normal right ventricular function.
- No signs of pulmonary hypertension or significant pericardial effusion.
- Minimal pericardial effusion.

Laboratory testing showed a peak high-sensitivity troponin T of 1182 ng/L, with progressive decline over the following days: 1102 ng/L, 233.1 ng/L, 190.5 ng/L, and 163.9 ng/L. C-reactive protein (CRP) was 0.03 mg/dL, erythrocyte sedimentation rate (ESR) was 8 mm/h, and renal and electrolyte panels were within normal limits. Autoimmune screening (ANA, ENA, ANCA) and infectious workup (EBV, CMV, SARS-CoV-2) were negative.

CMR was performed four days after symptom onset, using a Philips Ingenia 3.0T MRI system (Philips Healthcare, Best, The Netherlands), a digital broadband scanner equipped with dStream technology. The protocol included T2-weighted short tau inversion recovery (STIR) and phase-sensitive inversion recovery (PSIR) sequences optimized for myocarditis evaluation, with LGE performed 10 min after intravenous administration of gadolinium contrast (Dotarem, 0.3 mL/kg).

CMR findings included the following:

- Increased myocardial signal intensity on T2-STIR sequences (ratio 3:1), suggestive of myocardial edema.
- Subepicardial LGE in the lateral wall of the left ventricle, consistent with myocardial injury.
- Mild left ventricular systolic dysfunction (EF 45%).
- Left ventricular end-diastolic volume (LVEDV) of 132 mL (85.1 mL/m^2^).
- Left ventricular end-systolic volume (LVESV) of 73 mL (47 mL/m^2^).
- No significant valvular abnormalities.
- No evidence of pericardial inflammation or effusion.

**Figure 2:**
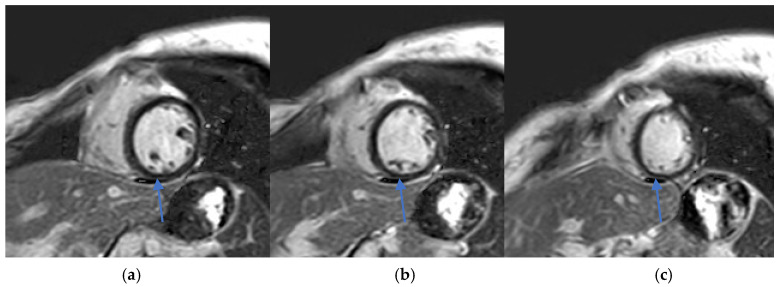
(**a**–**c**) Phase-sensitive inversion recovery (PSIR) sequence, short-axis view. Subepicardial late gadolinium enhancement (LGE) is visualized in the inferior wall (arrows), consistent with myocarditis.

**Figure 3:**
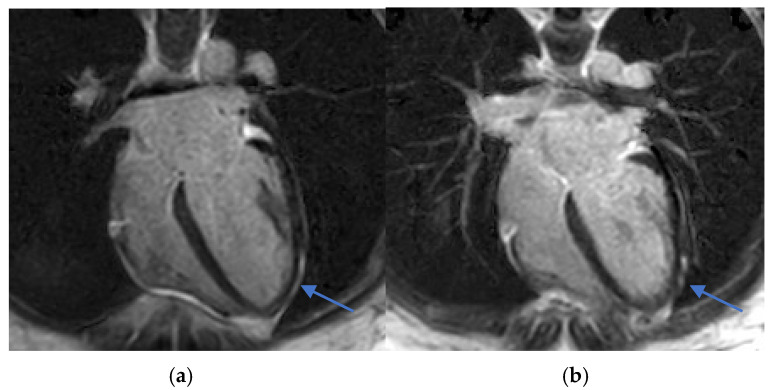
(**a**,**b**) Phase-sensitive inversion recovery (PSIR) sequence, 4-chamber view. Subepicardial late gadolinium enhancement (LGE) is seen in the lateral wall (arrows), supporting the diagnosis of myocarditis.

T1 and T2 mapping sequences were not acquired, which represents a limitation in tissue characterization. Nevertheless, these findings were consistent with acute myocarditis according to the 2018 revised Lake Louise criteria (Figure 2 and Figure 3).

### 2.3. Differential Diagnosis and Rationale

The differential diagnosis included acute coronary syndrome, viral myocarditis, pericarditis, stress-induced cardiomyopathy, and drug-induced myocarditis. Acute coronary syndrome was excluded by normal coronary anatomy on CTA and lack of ischemic ECG changes. Viral myocarditis was considered unlikely due to negative serologies and absence of systemic symptoms. Autoimmune causes were also unlikely given the normal inflammatory markers and negative serologies. The temporal association with ZA infusion, combined with the exclusion of other causes, supported a diagnosis of presumed ZA-related myocarditis, although histopathological confirmation was not obtained.

### 2.4. Management and Clinical Course

Upon diagnosis of presumed ZA-induced myocarditis, ZA was discontinued, and the patient was managed conservatively with close cardiac monitoring. Given the mild clinical course and favorable biomarker trend, no anti-inflammatory treatment was initiated. Her symptoms improved significantly within several days of admission. Follow-up CMR three months later demonstrated complete resolution of myocardial inflammation and recovery of left ventricular function (EF 50–55%). ZA rechallenge was avoided to mitigate the recurrence risk. The patient was informed about the suspected adverse event and advised to report it in future medical encounters.

## 3. Discussion

To our knowledge, this is the first reported case of presumed acute myocarditis temporally associated with zoledronic acid infusion, confirmed by CMR imaging. Although ZA is not typically linked to cardiotoxicity, there is growing evidence suggesting that it may exert immunomodulatory effects beyond its primary action on bone resorption [12,13,14,15]. Given ZA’s broad use in oncology and endocrinology, even rare immunologic cardiac complications raise significant concern, especially when diagnosis relies solely on imaging rather than histopathology.

### 3.1. Differential Diagnosis and Diagnostic Reasoning

A thorough and structured approach to differential diagnosis is essential in cases of suspected drug-induced myocarditis. In our patient, the clinical presentation, including chest pain and elevated troponin levels, prompted consideration of multiple potential etiologies. The initial differential diagnosis encompassed acute coronary syndrome, viral myocarditis, pericarditis, stress-induced cardiomyopathy, and drug-induced myocarditis.

A critical aspect of the diagnostic process involves systematically excluding common and more likely causes. Acute coronary syndrome was ruled out based on the patient’s anamneses and the absence of ischemic changes on ECG. Viral myocarditis, though a possible cause, was deemed less likely given the lack of respiratory or gastrointestinal symptoms and negative viral serologies. Similarly, autoimmune myocarditis was considered unlikely due to the absence of systemic inflammatory markers (CRP, ESR) and normal autoimmune panel results. Stress-induced cardiomyopathy was also considered, but the lack of a triggering psychological or physical stressor and the specific CMR findings were inconsistent with this diagnosis. Additionally, pheochromocytoma-induced myocarditis was considered, given the patient’s history, but was deemed less likely due to the stable endocrine management of her condition with somatostatin analog therapy, which tilted the diagnosis toward presumed ZA-induced myocarditis. The close temporal relationship between ZA administration and symptom onset, combined with the exclusion of other causes, suggests presumed ZA-induced myocarditis. Notably, these conclusions are based on exclusion in the absence of tissue diagnosis.

### 3.2. Mechanistic Discussion on the Pathophysiology of Drug-Induced Myocarditis

Drug-induced myocarditis is typically characterized by inflammatory infiltration of the myocardium, often initiated through immune activation or direct cytotoxicity by pharmacological agents. The mechanisms may include cytokine release, immune cell recruitment, endothelial injury, and inflammasome activation, all contributing to myocardial inflammation and injury [7,8,15]. In this context, zoledronic acid, a nitrogen-containing bisphosphonate, has been shown to influence immune pathways, potentially predisposing to myocarditis under susceptible conditions.

ZA primarily acts by inhibiting farnesyl pyrophosphate synthase within the mevalonate pathway, disrupting osteoclast-mediated bone resorption. However, inhibition of this pathway also interferes with intracellular signaling in immune cells, altering monocyte and macrophage function. This can promote a pro-inflammatory M1 phenotype and initiate activation of the NLRP3 inflammasome—a key innate immune sensor that detects cellular stress and infection [16,17]. NLRP3 activation leads to the release of interleukin-1β (IL-1β), tumor necrosis factor-alpha (TNF-α), and interleukin-6 (IL-6), cytokines that are directly implicated in the pathogenesis of myocarditis and other cardiovascular inflammatory disorders [18,19].

Although these mechanistic insights support a plausible immunoinflammatory basis for ZA-associated myocarditis, direct experimental or clinical evidence remains limited. Most data are derived from preclinical or observational studies and extrapolations from related inflammatory syndromes. Further research, including prospective studies and animal models, is necessary to elucidate the specific immunologic triggers and patient-related risk factors (e.g., underlying inflammatory disease, prior cardiotoxic therapy) that may increase susceptibility to myocarditis following ZA administration [20,21].

### 3.3. Diagnostic Approach and Utility of CMR

**Figure 4:**
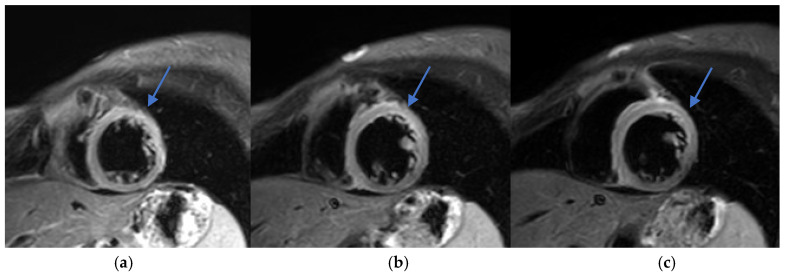
(**a**–**c**) T2-weighted short tau inversion recovery (T2-STIR) images. Increased signal intensity is noted in the anterior wall (arrows). The myocardium-to-skeletal muscle signal intensity ratio is approximately 3:1, consistent with myocardial edema.

Early recognition and accurate diagnosis of myocarditis are critical to initiating timely management and preventing potential long-term cardiac sequelae. Diagnosing drug-induced myocarditis, especially when triggered by bisphosphonates like ZA, poses significant challenges due to nonspecific symptoms and the wide range of potential causes. In this case, CMR was pivotal, showing increased myocardial signal intensity on T2-STIR sequences (signal intensity ratio 3:1) and subepicardial late gadolinium enhancement in the lateral wall of the left ventricle (Figure 2, Figure 3 and Figure 4). These findings are consistent with acute myocarditis as defined by the 2018 revised Lake Louise criteria [22].

Although T1 and T2 mapping was not performed, precluding quantitative tissue characterization, T2-STIR still provided meaningful visual evidence of edema. While the technique has known limitations—such as motion artifacts and lower spatial resolution—it remains useful in the clinical setting. Notably, no inferior wall subepicardial LGE was observed, reinforcing a non-ischemic distribution consistent with myocarditis rather than infarction.

The diagnostic advantages of CMR in this context include the following:

1. Non-invasive and accurate: Enables visualization of myocardial inflammation and injury without the need for endomyocardial biopsy, which has low sensitivity and procedural risks [23].
2. High sensitivity for subepicardial lesions: Particularly effective in detecting non-ischemic myocardial injury patterns that might not be apparent on echocardiography or ECG [23].
3. Impact on management: In this case, CMR findings led directly to the discontinuation of ZA and initiation of supportive care, exemplifying how imaging can inform critical therapeutic decisions.

As such, CMR remains the cornerstone imaging modality for diagnosing myocarditis, especially in cases with ambiguous clinical presentations or suspected drug-induced etiologies [24].

### 3.4. Comparison with Other Bisphosphonates and Immune Checkpoint Inhibitors

While ZA is the focus of this report, the possibility of drug-induced myocarditis from other pharmacological agents deserves consideration. Denosumab, a monoclonal antibody targeting RANKL used for osteoporosis and metastatic bone disease, has been associated with systemic inflammatory syndromes in rare instances. However, no robust clinical or preclinical evidence currently links denosumab to myocarditis or direct cardiac inflammation in humans, and cardiac involvement remains undocumented in the literature [25].

In contrast, ICIs, such as those targeting programmed death-1 (PD-1) and cytotoxic T-lymphocyte-associated protein 4 (CTLA-4), have a well-documented association with myocarditis as a serious immune-related adverse event (irAE). ICI-induced myocarditis is thought to be mediated by aberrant T-cell activation, resulting in myocardial infiltration, necrosis, and fibrosis. Though relatively rare, this condition carries a high mortality rate and often presents early in the course of treatment [10].

Compared to the ICI-associated myocarditis paradigm, the association between ZA and myocarditis remains largely theoretical and based on rare case reports or mechanistic hypotheses. Table 1 outlines key differences in pathophysiology, clinical presentation, and management strategies among ZA, denosumab, and ICIs. While ICIs have a clear immunopathogenic mechanism, ZA’s role remains speculative and potentially mediated by inflammasome activation or γδ T-cell stimulation, as previously discussed.

### 3.5. Literature Review of Similar Reported Cases

Only a few reports of ZA-associated myocarditis exist in the literature. A notable case by Watts et al. documented acute myopericarditis in a patient treated with intravenous ZA for Paget’s disease, with complete resolution of symptoms following drug discontinuation [9]. Another case described a sepsis-like syndrome following ZA infusion, suggesting robust systemic immune activation as a plausible mechanism [18]. Although these cases underscore the potential for ZA-induced inflammatory responses, histopathological confirmation was absent, and causality remains speculative. The extreme rarity of CMR-confirmed cases—such as the one reported here—emphasizes the need for continued vigilance and reporting.

### 3.6. Clinical Implications for Monitoring and Managing ZA-Induced Myocarditis

With the expanding use of ZA in oncology and metabolic bone disease, clinicians must remain vigilant for atypical presentations, including myocarditis. While routine cardiac screening is not warranted for asymptomatic individuals, a high index of suspicion is appropriate in patients presenting with unexplained chest pain, dyspnea, or elevated cardiac biomarkers after infusion. The temporal relationship with ZA administration—particularly when other etiologies are excluded—should prompt early cardiac evaluation.

Timely diagnosis and cessation of ZA, combined with supportive care, can lead to full clinical recovery, as seen in this case. This outcome reinforces the importance of recognizing early warning signs and acting decisively. Furthermore, the potential need for risk stratification—particularly in patients with known inflammatory conditions or prior exposure to cardiotoxic agents—deserves exploration in future studies.

### 3.7. Limitations

This case report is inherently limited by the absence of endomyocardial biopsy, which was not pursued due to the patient’s stable clinical status and favorable outcome, thereby precluding histopathological confirmation of myocarditis. As a single case, the report cannot establish causality, and the temporal association between zoledronic acid and myocardial inflammation remains presumptive. Additionally, the lack of advanced tissue characterization through T1 and T2 mapping sequences on cardiac CMR restricts the granularity of myocardial tissue assessment and limits diagnostic precision.

## 4. Conclusions

This case illustrates the potential for acute myocarditis as a rare but clinically relevant complication temporally associated with ZA infusion. The diagnosis was supported by the patient’s acute presentation, elevated cardiac biomarkers, and characteristic CMR findings—including subepicardial late gadolinium enhancement—though lacking histopathological validation.

While ZA is primarily indicated for the prevention of skeletal-related events in patients with metastatic bone disease and other osteolytic conditions, emerging evidence suggests a potential for rare systemic immune activation, including cardiac involvement.

Clinicians should maintain a high index of suspicion for myocarditis in patients presenting with unexplained chest pain shortly after ZA administration, particularly when other common causes (ischemic, infectious, or autoimmune) are excluded. Although pheochromocytoma-related myocarditis was a possible confounder in this case, the patient’s stable disease and the close temporal relationship with ZA administration favored a presumed drug-related etiology.

CMR proved to be a critical diagnostic tool in this setting, enabling non-invasive identification of myocardial inflammation and informing treatment decisions. The imaging findings were consistent with acute myocarditis, with LGE patterns more suggestive of necrosis than chronic fibrosis.

Importantly, while endomyocardial biopsy remains the gold standard for definitive myocarditis diagnosis, it is not routinely recommended in stable cases without life-threatening arrhythmias, heart failure, or hemodynamic compromise. In such scenarios, CMR serves as a non-invasive and highly informative diagnostic modality. Recent expert consensus guidelines emphasize that CMR—particularly when interpreted via the updated Lake Louise criteria—can reliably establish the diagnosis of myocarditis in low- to intermediate-risk patients, reducing the need for biopsy in appropriate clinical contexts [23,24].

Looking forward, research efforts should focus on identifying patient-specific risk factors that may predispose to ZA-related cardiac inflammation. A systematic approach to pharmacovigilance, including registry-based reporting of suspected cardiac adverse events, is necessary to clarify incidence, risk stratification, and pathophysiology.

Finally, consideration should be given to potential contributing factors—such as neuroendocrine tumors or previous cardiotoxic exposures—in evaluating future cases. Predictive models incorporating patient comorbidities, immune profiles, and imaging markers may enhance clinical decision-making regarding ZA use in high-risk individuals.

In summary, this case underscores the importance of early recognition, careful differential diagnosis, and the central role of CMR in evaluating suspected myocarditis following ZA infusion. It emphasizes the need for individualized risk assessment and timely intervention to ensure patient safety while preserving therapeutic benefit.

## Figures and Tables

**Table 1:** Comparing drug-induced myocarditis cases from the literature.

DRUG/AGENT	MECHANISM	CLINICAL PRESENTATION	MANAGEMENT
ZOLEDRONIC ACID	NLRP3 inflammasome activation, cytokine release	Chest pain, elevated troponin, CMR-positive myocarditis	Discontinuation, supportive care
DENOSUMAB	Potential cytokine modulation	Inflammatory syndrome	Discontinuation
ICIS	T-cell activation, myocarditis as irAE	Severe myocarditis, arrhythmias	Discontinuation, immunosuppressive therapy

## Data Availability

The original contributions presented in this study are included in the article. Further inquiries can be directed to the corresponding author(s).

## Abbreviations

The following abbreviations are used in this manuscript:

ZA	Zoledronic acid
CMR	Cardiac magnetic resonance imaging
LGE	Late gadolinium enhancement
TTE	Transthoracic echocardiography
ECG	Electrocardiography
TNF-α	Tumor necrosis factor-alpha
IL-1β	Interleukin-1 beta
IL-6	Interleukin-6
NLRP3	Nucleotide-binding domain, leucine-rich repeat, pyrin domain-containing protein 3
EBV	Epstein–Barr virus
CMV	Cytomegalovirus
ANA	Antinuclear antibody
ENA	Extractable nuclear antigen
ANCA	Antineutrophil cytoplasmic antibody
CRP	C-reactive protein
ESR	Erythrocyte sedimentation rate
PSIR	Phase-sensitive inversion recovery
DOPA PET/CT	Dihydroxyphenylalanine positron emission tomography/computed tomography
ICIs	Immune checkpoint inhibitors
irAE	Immune-related adverse event
EF	Ejection fraction
